# Transport of cerium oxide nanoparticles in saturated silica media: influences of operational parameters and aqueous chemical conditions

**DOI:** 10.1038/srep34135

**Published:** 2016-10-03

**Authors:** Zhaohan Zhang, Peng Gao, Ye Qiu, Guohong Liu, Yujie Feng, Mark Wiesner

**Affiliations:** 1 State Key Laboratory of Urban Water Resource and Environment, Harbin Institute of Technology, No73, Huanghe Road, Nangang District, Harbin 150090, China; 2 Center for the Environmental Implications of Nano Technology, Duke University, Durham, North Carolina 27708, United States

## Abstract

This paper aimed to investigate the influences of operational parameters and aqueous chemical conditions on transport behaviors of cerium oxides nanoparticles (CeO_2_-NPs) in saturated silica media. Results indicated that increasing rates of attachment efficiency (α) were related with cationic types, and critical deposition concentration (CDC) for divalent cation (Ca^2+^ and Mg^2+^) were more than 31-fold of that for monovalent cation (Na^+^ and K^+^). Increase or reduction of electrolyte pH could both promote the mobility of CeO_2_-NPs in glass beads, while influence was more evident at alkaline conditions. α increased linearly with NPs concentrations, while decreased linearly with flow velocity in the column, and effects were related with electrolyte contents. Presence of surfactants could sharply decreased α, and SDS was more effective to facilitate CeO_2_-NPs transport than Triton X–100. With DOMs concentrations increasing, α firstly kept constant, then sharply declined, and finally reduced very slowly. The influence of DOMs on NPs deposition was in order of SA > HA > TA >  BSA. Overall, this study revealed that aqueous chemical conditions was crucial to NPs transport in porous media, and would provide significant information for our understanding on the fate and transport of nanoparticles in natural environment.

CeO_2_-NPs are currently used in a various industrial, agricultural and consumer application, including catalytic converters, diesel fuel additive, gas sensors, solar cells, solid fuel cells, UV blocking agent and polishing agents^1^,^2^,^3^. Global production of CeO_2_-NPs was estimated at approximately 10000 tons/year, of which 1% ended in air, 4% in water and 14% in soil, and it had been listed as one of the 13 representative manufactured nanomaterials^4^. Due to the prevailing productions and uses, CeO_2_-NPs were inevitably released into aquatic and terrestrial environments^5^. The estimated CeO_2_-NPs concentrations in STP effluent were in range of 0.003–1.17 μg/L, while much higher levels (0.53–9.1 mg/kg) were expected in associated biosolids^6^,^7^. Although these concentrations were in lower levels, they also might pose a potential risk to human health and ecological safety.

CeO_2_-NPs was demonstrated to be toxic toward bacteria, plants, zebra fish, soil nematodes, algae, epithelial and cancer of human lung^8^,^9^. It was proposed that toxicity mechanisms included oxidative stress, reactive oxygen species production(ROS), cell membrane damage, lipid peroxidation, release of free Ce(III), substantial bioaccumulation in liver of zebra fish, disturbance in plant defense mechanisms or the rise of intracellular ROS caused by direct contact between cells and nanoparticles^8^,^10^,^11^,^12^. Moreover, aqueous chemical condition could change nanoparticles toxicity. Collin *et al*. found that addition of humic acid (HA) to exposure media evidently decreased toxicity of CeO_2_-NPs and ratio of CeO_2_-NPs to HA influenced Ce bioaccumulation^13^. Presence of surfactants in NPs exhibited two different joint effects on organism: (1) strengthen the dispersibility and toxicity by altering surface charge of NPs, and (2) reduce the toxicities by inhibiting interaction between NPs and bacteria through steric hindrance and charge repulsion^14^. Most studies reported that natural organic matter (NOM) reduced the toxicity of nanoparticles to organisms^15^,^16^. DOM could stabilize nanoparticles through the combination of van der Waals forces, electrostatic forces and steric effects, which was further influenced by various factors, such as molecular structure and weight, pH, ionic strength, charge, ions valence, temperature, etc^17^. Therefore, aqueous chemical condition was crucial to toxicity of CeO_2_-NPs in natural environment.

Due to toxicity and potential ecological risk of CeO_2_-NPs on humans and ecosystem, it is important to improve our understandings on transport and fate of CeO_2_-NPs in natural environment. Up to now, several researchers have investigated the transport behaviors of CeO_2_-NPs in porous media. Li *et al*. reported that CeO_2_-NPs was easily transported in porous media at neutral and alkaline conditions, and almost deposited in sand column with the ionic strength increasing to 10 mM or pH decreasing to 3^18^. Hassan *et al*. developed a simulation applicable to quantitatively investigating the influence of process conditions and operating parameters on CeO_2_-NPs transportation, and found that steady state flow of NPs was not retained by a sand filter, while spike concentrations could be dampened effectively^19^. Previous studies also found that presence of low levels of Suwannee River humic acid, fulvic acid, citric acid, alginate and carboxymethyl cellulose could evidently enhance the stability and mobility of CeO_2_-NPs^20^,^21^. Cornelis *et al*. proposed that low retention of CeO_2_-NPs in soil was associated with naturally occurring colloids, such as Al, Si and Fe oxides^22^. Petosa *et al*. observed that polyacrylic acid coated CeO_2_-NPs were more easily transport in quartz sand column by comparison with loamy sand column^23^. Fang *et al*. found that CeO_2_-NPs mobility was significantly enhanced by the macropore in quartz sand and soil, and enhancement was greater in continuous macropore than in discontinuous one^24^. However, few studies have systematically investigated the impacts of cationic types, surfactants and dissolved organic matters in a large concentration range on transport behavior of CeO_2_-NPs in porous media.

To better understand CeO_2_-NPs transport and fate in groundwater or engineered processes of filtration, it is crucial to clarify the physical and chemical principles of nano-particles transport through porous media, especially at different operational parameters and aqueous chemical conditions. However, relevant information is scarce. The main objectives of present study are to determine (1) the effects of cationic types and strengths, pH, initial concentrations and flow rates on retention and transport of CeO_2_-NPs in saturated porous media, and (2) the roles of surfactants and four dissolved organic matters in deposition of CeO_2_-NPs on media surfaces. The results of this study are expected to provide us some important insights on better understanding the transport, fate and relative mobility of nanoparticles in natural soil or contaminated ground water, and evaluating the potential exposure and environmental risk of nanoparticles.

## Results and Discussion

### Effect of cationic type and strength

**Figure 1:**
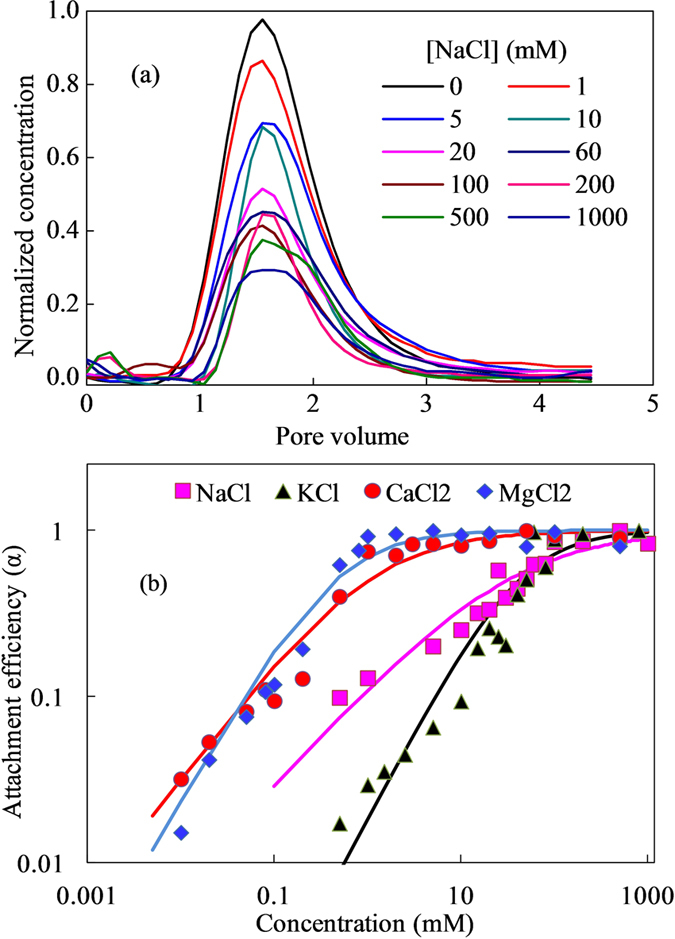
Illustrative transport results and critical deposition curves of CeO_2_-NPs at varying NaCl concentration (**a**) and four different cationic solutions (**b**). (**a**) Were normalized column effluent NPs concentrations versus number of pore volumes for washing solution at pH of 5.54. (**b**) were obtained at pH 5.54, injection of 1 mL 100 mg/L NPs, and flow rate of 2.0 mL/min.

Four common cationic (Na^+^, K^+^, Ca^2+^ and Mg^2+^) with different valence states were selected to investigate the effects of ionic type and strength on NPs transport. Taken NaCl as an example, breakthrough curves of CeO_2_-NPs at column outlet with different NaCl are presented in Fig. 1(a). Normalized NPs concentrations in effluent were plotted with number of pore volumes. It had a peak at approximately 1.5 times pore volumes. Significantly, the mobility of CeO_2_-NPs in glass bead is high, attributing to the good stability of suspension. Without NaCl addition, almost 95% of NPs was eluted from column. NaCl addition could enhance deposition and aggregation on glass beads, with 88.4%, 70%, 42.7% and 34.5% of NPs eluting from column for NaCl concentration of 1, 10, 100 and 1000 mM, respectively. With ionic strength increased, diffuse double layers were compressed resulting in a reduction in repulsive electrostatic double-layer forces and attractive van der Waals forces were dominant, leading to increased deposition and aggregation rate of NPs^25^. The other three cationic also had the similar phenomenon.

Attachment efficiency at different cationic contents was shown in Fig. 1(b). All deposition curves were separated into unfavorable and favorable region by CDC. In unfavorable region (below CDC), mass recovery ratio decreased rapidly with cationic concentration increase. A 10-fold increase in cationic concentration resulted in approximately 2.3, 5.1, 4.4 and 7.3-fold increases in α for electrolyte of NaCl, KCl, CaCl_2_ and MgCl_2_, respectively, indicating that increasing rate of α was closely related to cationic type. Moreover, it also varied significantly with different nanoparticles. Jaisi *et al*. observed that a 10-fold increase in ionic strength only produced 2-fold increase in α of single-walled carbon nanotubes at unfavorable region^26^. According to DLVO theories, as the cation concentration increased, the electrostatic energy barrier was reduced, which promoted aggregation and resulted in the gradual increase of attachment efficiency. As the cation concentration reached the CDC, the energy barrier was completely eliminated and the attachment efficiency was close to unity, from which the aggregation fell into the favorable region. In this region, mass recovery ratios reduced to minimum and kept almost constant with cationic concentration continued to increase. The minimum recovery corresponded to a maximal deposition rate with α = 1, indicating that deposition in this section was only controlled by diffusion and mass transfer process, while irrelevant to electrolytic characteristics and concentration. CDC of NaCl, KCl, CaCl_2_ and MgCl_2_ were calculated as 31.6, 42.7, 0.45 and 1.02 mM, respectively. At pH 5.54, CeO_2_-NPs were positively charged and Cl^−^ was counter ion, but CDC for divalent cation (Ca^2+^ and Mg^2+^) were more than 31-fold of that for monovalent cation (Na^+^ and K^+^). The higher deposition in presence of divalent cation could be explained by formation of divalent cation bridging as described in previous studies^27^,^28^. They reported that complexation of Ca^2+^ or Mg^2+^ ions to carboxylic acid on NPs and silanol groups on collector surface produced localized nano-scale heterogeneities being favorable for deposition. Moreover, it could also be explained by effect of valence on Debye length and in turn the effect of Debye length on electrostatic repulsion^29^. Due to the inverse of Debye length was proportional to the valence charge of ion, so Debye length decreased with valence charge, resulting in a lower magnitude electrostatic repulsive energy which likely promoted NPs aggregation and deposition. Our results were in agreement with the observations from Saleh *et al*. on the effects of ionic strength and composition on zerovalent iron nanoparticles^30^.

### Effect of pH

**Figure 2:**
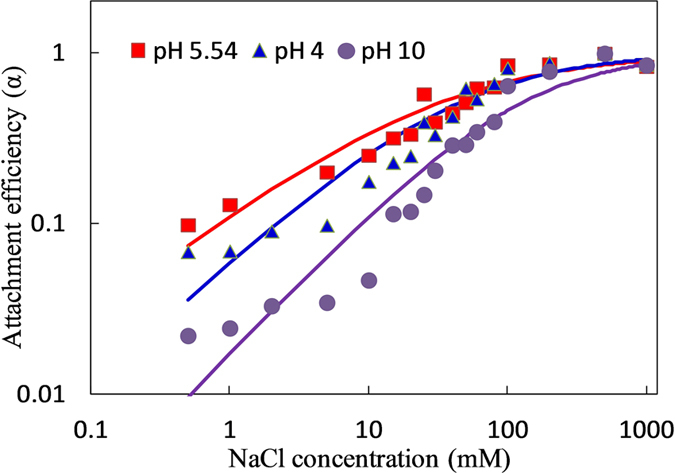
The critical deposition curves of CeO_2_-NPs at pH of 4, 5.54 and 10 with NaCl as the electrolyte. 100 mg/L CeO_2_-NPs was pulsing injected into the transport column at 1 mL.

Effect of pH on CeO_2_-NPs transport in porous media was studied with NaCl as electrolyte. As shown in Fig. 2, all deposition curves could be fitted by equation (5) with R^2^ >0.863, and separated into three segments. The attachment efficiency slowly increased up to 10 mM NaCl, rapidly increased between 10–100 mM NaCl, and eventually reached to a constant after 100 mM NaCl. At low NaCl concentrations (<10 mM), repulsive forces dominated and very little aggregation occurred; while at higher concentrations (10–100 mM), particle aggregation rate increased with NaCl concentration due to the reducing of repulsion barrier; at the highest NaCl concentrations(>100 mM), attractive van der Waals forces dominated over repulsive forces and highest aggregation rate were obtained. The calculated CDC values were 43.1, 31.6 and 122.6 mM at pH of 4.0, 5.54 and 10.0, respectively. It indicated that increase or reduction of pH in electrolyte both promote the mobility of NPs in glass beads. Liu *et al*. reported that the retention of CeO_2_ on sand was reduced from 99.2% to 94.3% with pH increasing from 4.0 to 8.5, and most NPs deposited at the first 1 cm of sand from the inlet^20^. While Li *et al*. found that transport was significantly hindered at acidic conditions (pH 3) and high ionic strengths (10 mM and above), and the deposited CeO_2_ particles might not be re-entrained by increasing the pH or lowering the ionic strength of water^18^. The difference might be related to media types and surface charge. A 10-fold increase of NaCl concentration led to approximately 2.8, 2.3 and 8.2-fold increases in α at pH of 4.0, 5.54 and 10.0, indicating that influence of electrolyte on NPs mobility were more evident at alkaline conditions. At pH 10, both NPs and collectors were strongly negatively charged. Electrostatic repulsion was dominated in this condition, resulting in a higher mobility of NPs in porous media, which needed higher concentration of electrolyte to promote deposition. With pH decreasing, higher proton concentration appeared in solution, which could react with negatively charged functional groups, such as carboxyl groups on NPs and silanol groups on glass beads, leading to a reduced surface charge on both NPs and collectors. Based on DLVO theory, barriers would be reduced to benefit for attachment. However, when pH decreased to lower than pH_PZC_ (point of zero charge), NPs surface became positively charged. It was found that pH_PZC_ of CeO_2_-NPs in pure water was 6.5, which was close to the theoretical value of pH_PZC_ = 7 31. At this condition, electrostatic attraction was predominated. Therefore, at pH 5.54 (closing to pH_PZC_), NPs with a little positive charge were favorable to deposition and aggregation with negative charged glass beads. At much lower pH, NPs surface and collectors were both strongly positively charged, resulting in electrostatic repulsion re-predominated. Moreover, lower pH could also dissolve some CeO_2_-NPs, which also increased its mobility in porous media.

### Effect of initial CeO_2_-NPs concentration

**Figure 3:**
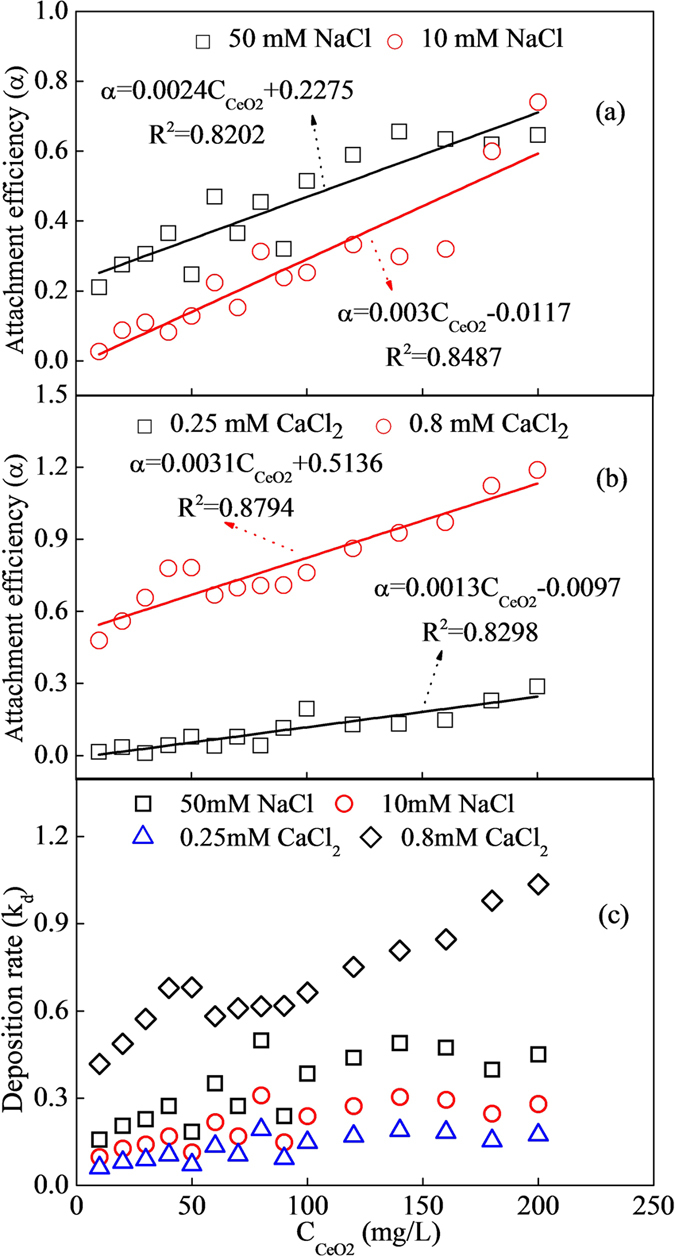
Attachment efficiency and deposition rate of CeO_2_-NPs for different nanoparticle concentrations (10–200 mg/L) in NaCl and CaCl_2_ at the flow rate of 2.0 mL/min. (**a**) α changed with CeO_2_-NPs conentration with NaCl of 10 and 50 mM as electrolyte; (**b**) α changed with CeO_2_-NPs conentration with CaCl_2_ of 0.25 and 0.8 mM as electrolyte; (**c**) k_d_ changed with CeO_2_-NPs conentration with NaCl and CaCl_2_ as electrolyte.

Influence of NPs content on mobility was evaluated at 15 different injection concentrations ranging from 10 to 200 mg/L with NaCl (10 and 50 mM) and CaCl_2_ (0.25 and 0.8 mM) as background electrolyte. As shown in Fig. 3, α in four electrolytes all increased linearly with CeO_2_-NPs concentrations. Growth rates for α were estimated as 0.003 and 0.0024 for NaCl (10 and 50 mM), 0.0013 and 0.0031 for CaCl_2_ (0.25 and 0.8 mM), respectively, demonstrating that influences of NPs on deposition were evidently depending on cationic types and concentrations. When injection concentration (C_0_) increased from 10 to 200 mg/L, for NaCl, relative concentration (C/C_0_) decreased from 0.97 to 0.45 at 10 mM, while it decreased from 0.78 to 0.48 at 50 mM; for CaCl_2_, C/C_0_ were decreased from 0.95 to 0.38 at 0.25 mM, while it decreased from 0.51 to 0.19 at 0.8 mM. These demonstrated that the fraction of injected CeO_2_-NPs mass recovered in effluent generally decreased with C_0_ increasing at the same ionic strength, while it was much higher at higher ionic strength for the same C_0_. Deposition rate was in order of 0.8 mM CaCl_2_ >50 mM NaCl >10 mM NaCl >0.25 mM CaCl_2_ for all studied NPs concentrations. K_d_ were all increased with NPs in range of 10–140 mg/L, and then almost kept constant at 0.95 ± 0.10, 0.44 ± 0.04, 0.27 ± 0.02 and 0.17 ± 0.01 up to 200 mg/L, indicating that higher NPs concentration benefiting for its deposition. Zhang *et al*. also reported the similar result that greater C_0_ (10–1000 mg/L) could decrease relative colloidal efflux and increase deposition rate coefficient at IS >0.1 mM due to retention sites growing faster at greater C_0_^32^. Rahman *et al*. found that increased and faster elution was observed when the influent concentration of aluminum oxide nanoparticles was increased from 5 mg/L to 400 mg/L in the column transport experiments^33^. When NPs were injected into glass bead column, they firstly deposited on surface of glass bead. Due to energy barriers between CeO_2_-NPs and CeO_2_-NPs were much lower than those between CeO_2_-NPs and glass bead, previous deposited NPs would provide new retention sites for the following NPs. At higher C_0_, the number of firstly deposited CeO_2_-NPs was much higher than those at lower C_0_, resulting in retention sites increased with C_0_. However, too high C_0_ might enhance the blocking/filling of potential retention locations, resulting in deposition ratios not always increased with NPs concentrations^34^. These concentration effects on NPs transport were closely related to the characteristics of both NPs and collectors, especially characteristics affecting the energy barriers between CeO_2_-NPs-CeO_2_-NPs or CeO_2_-NPs-collectors, such as surface potentials, surface geometry, particle size, hydrodynamic force and Hamaker constant, etc.

### Effect of injection flow rate

**Figure 4:**
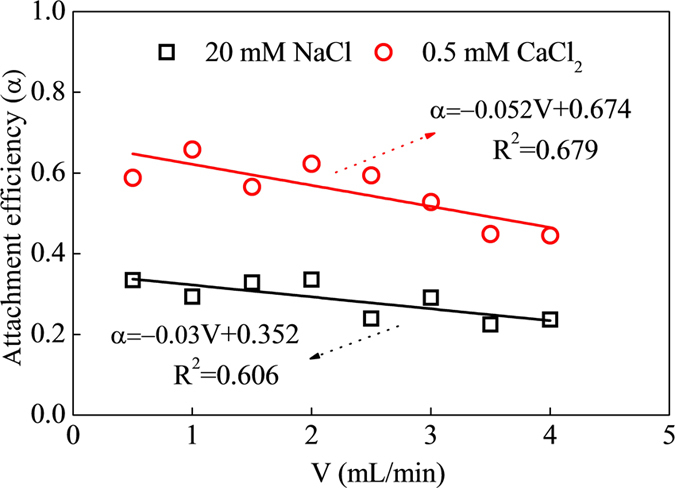
Attachment efficiency of CeO_2_-NPs at eight different flow rates (0.5–4.0 mL/min). Results were obtained at pH of 5.54, injection of 1 mL 100 mg/L NPs, with 20 mM NaCl and 0.5 mM CaCl_2_ as electrolyte.

To study effects of flow rate on mobility, NPs were passed through column at eight different flow rates in range of 0.5–4.0 mL/min with 100 mg/L CeO_2_-NPs injection in 20 mM NaCl and 0.5 mM CaCl_2_. As shown in Fig. 4, α both decreased linearly with increasing velocity in the column, with reducing rates of 0.03 at 20 mM NaCl and 0.052 at 0.5 mM CaCl_2_, indicating that higher flow rate was benefited to NPs mobility and this effect was related to electrolyte concentration. Sasidharan *et al*. reported the similar phenomenon that the delay in breakthrough curves became shorter and NP deposition decreased with increasing flow velocity^35^. While Rahman *et al*. reported that varying flow conditions in range of 0.21–2.55 mL/min did not have a significant effect on mobility of aluminum oxide nanoparticles in saturated sand^33^. The results were consistent with prediction of colloid filtration theory, that decreasing flow rate in a porous medium increased the number of collisions occurring between a passive colloid and particles and promote the colloid retention in the media^36^. These trends could be explained partially by torque balance considerations. The hydrodynamic torque acting on NPs adjacent to solid surface became smaller as flow rate decreased, and produced a higher fraction of solid surface area available for NPs deposition. Ko and Elimelech attributed this observation to larger shadow areas down gradient of deposited particles and protrusions on collectors, based on the assumption that shadow regions were not accessible for NPs deposition at higher flow rate. Moreover, lower flow rate corresponded to a higher residence time, and it was assumed to increase of the adhesion strength, resulting in higher deposition of NPs^37^. Xu *et al*. confirmed that the adhesion force between microsphere and glass surface increased with residence time in range of 0.001–50 s by using colloid probe atomic force microscopy^38^. It might be ascribed to rearranging functional groups on surface of NPs for bridge and then binding to opposite surface until reaching the lowest energy state of all bonds.

### Effect of surfactants

**Figure 5:**
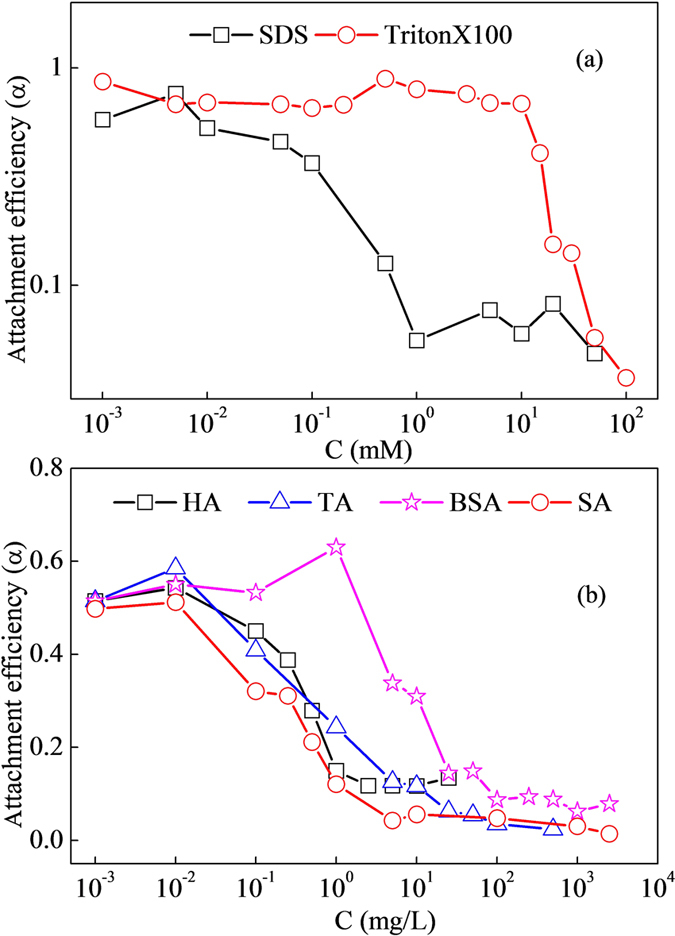
Influence of two surfactants (a) and four DOMs (b) on CeO_2_-NPs transport in glass beads columns. Conditions (**a**): 1 mL/min 100 mM NaCl + 1 mL/min surfactant solution, injection of 100 mg/L CeO_2_ 1 mL, with pore volume 3.22 mL. Conditions (**b**): 1 mL/min 50 mM NaCl + 1 mL/min organic solution, injection of 100 mg/L CeO_2_ 1 mL, with pore volume 3.22 mL.

To investigate the effectiveness of surfactant type on CeO_2_-NPs transport through glass beads, frequently-used anionic (SDS) and nonionic (Triton X-100) surfactant were compared with 50 mM NaCl as background electrolyte (Fig. 5a). It was obvious that CeO_2_-NPs transport was surfactant type dependent. With surfactant concentration increasing, α was first kept constant, then sharply decreased and finally kept almost constant at very low levels. For SDS, α became decreasing at 0.05 mM, and kept at approximate 0.065 ± 0.014 above 0.5 mM. However, for Triton X-100, α was kept 0.73 ± 0.08 before 10 mM, and quickly reduced to 0.038 at 100 mM. These results suggested that SDS was more effective to facilitate CeO_2_-NPs transport than Triton X-100 in our experimental conditions. Hua *et al*. found that SDBS was more effective than Triton X-100 to promote leaching of anionic pesticide, providing supports for the present results^39^. Due to surfactants addition could mask certain surface heterogeneity of both CeO_2_-NPs and glass beads, the favorable deposition sites would eliminate and extent of fast deposition at first stage would also reduce, resulting in NPs blocking effects elimination. Moreover, compared to Triton X-100, SDS as anionic surfactant, would make the NPs and glass beads surface more negative, consequently enhanced the repulsion force between NPs-NPs, and NPs-collectors, promoting the mobility of NPs in column. Godinez *et al*. also found the presence and concentration increase of surfactant enhanced the transport of nano-TiO2 in saturated porous media^40^. While Sun *et al*. reported that surfactants decreased the mobility of nano-TiO2 in soil columns and the inhibiting ability of different surfactants followed the order of SDS >Triton X-100. They thought the strong adsorption of surfactants on soil and nano-TiO2 was mainly responsible for the decrease of mobility^41^. The difference was mainly caused by the types and surface characteristics of medium and nanoparticles.

### Effect of organic matters

Four dissolved organic matters (BSA, HA, TA and SA), ubiquitously presenting in natural waters, were selected to test their influences on CeO_2_-NPs mobility. As shown in Fig. S1, normalized effluent CeO_2_-NPs concentrations (C/C_0_) were plotted as a function of pore volumes at different BSA and HA contents in 50 mM NaCl. NPs deposition both decreased (C/C_0_ increased) with increasing BSA and HA concentrations, indicating that presence of organic matters was facilitate the transport of CeO_2_-NPs through glass beads. The maximum of C/C_0_ was found as 0.94 and 0.89 in presences of 1000 mg/L BSA and 10 mg/L HA, which was 1.7 and 1.6 times as those control one (without DOM), while deposition rates were reduced by 88.9% and 81.4%, respectively.

α values obtained through above experiments presence of four different DOMs were plotted with DOMs concentrations in Fig. 5b. For all four DOMs, with DOMs concentrations increasing, α firstly kept constant, then sharply declined, and finally reduced very slowly. However, concentration range for each stage was different. For HA, TA and SA, at very low concentration (<0.01 mg/L), due to DOMs addition caused almost no change of solution and NPs characteristics, α was observed as no difference with the control one. When their concentrations were in range of 0.01–2.5, 0.01–25 and 0.01–5 mg/L, α sharply decreased from 0.54, 0.58 and 0.51 to 0.12, 0.06 and 0.04, reducing by 77.8%, 89.7% and 92.2%, respectively. With concentration continuously increasing, α for HA kept approximately at 0.12, while α for TA and SA were slowly decreased to 0.02 and 0.01 at 500 and 2500 mg/L. For BSA, the sharply decreasing stage was located in the range of 1–100 mg/L, and α was declined from 0.63 to 0.09, reducing by 85.7%. It was kept at about 0.08 thereafter. These results indicated that deposition was sensitive to DOMs in a certain concentration rage. By comparison, influence ability of DOMs on NPs deposition was in order of SA > HA > TA > BSA, which was related to the structure and characteristics of DOMs. Rapid drop of α at lower DOMs concentration (<5 mg/L) might be caused by monolayer coverage of all available CeO_2_-NPs surfaces. Due to injection of CeO_2_-NPs was 100 μg and its surface area was 115 m^2^/g based on BET method, occupied area for one milligram of SOM was estimated at 0.71 m^2^. It was lower than value of 1 m^2^ which was estimated as the area for complete monolayer coverage on a mineral surface by organic molecules^42^. Moreover, DOMs addition might also compete with CeO_2_-NPs for attachment sites on glass bead surface. At higher DOMs concentration, adsorbed layer of DOMs on NPs and collectors became thickening and would block CeO_2_-NPs surfaces to steric interaction, which would change surface charge more negative and made electrostatic repulsion as the dominant mechanism, resulting in the lower sensitivity of α at higher DOMs.

**Figure 6:**
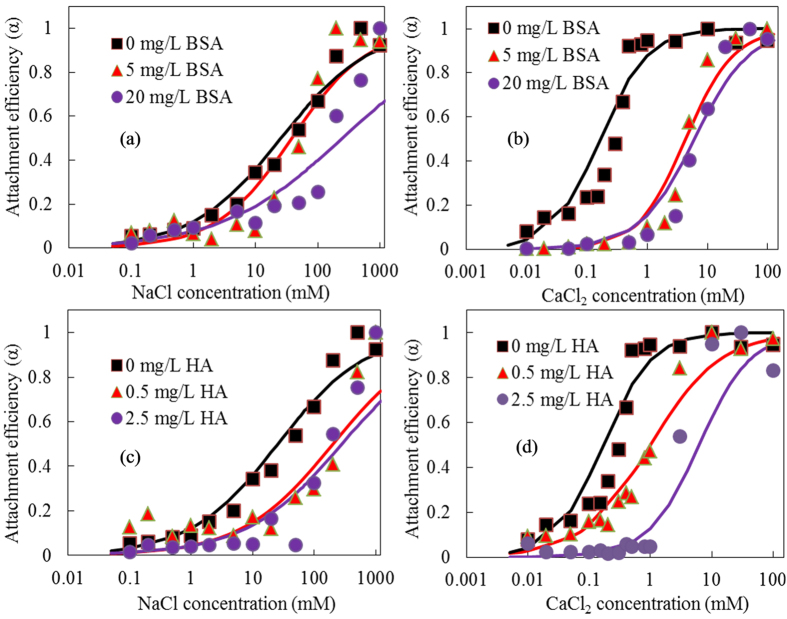
Critical deposition curves of CeO_2_-NPs with varied BSA (**a,b**) and HA (**c,d**) concentrations at pH 5.54 in presence of NaCl (**a,c**) and CaCl_2_ (**b,d**) as background electrolytes. The BSA concentration was selected at 5 and 20 mg/L, while HA concentration was selected at 0.5 and 2.5 mg/L.

BSA and HA were selected to investigate influence of DOMs contents on critical deposition curves with NaCl and CaCl_2_ as background electrolytes. For NaCl-based experiments, CDC curves for BSA (0, 5 and 25 mg/L) and HA (0, 0.5 and 2.5 mg/L) at pH 5.54 were shown in Fig. 6a,c. By fitting these stability curves with equation (5), CDC values were estimated as 44 and 282 mM NaCl for 0.5 and 2.5 mg/L HA, 38.4 and 249 mM NaCl for 5 and 20 mg/L BSA, which were 1.71, 10.9, 1.4 and 9.7 of that without DOM addition, respectively. It suggested that addition DOMs could promote mobility of NPs in porous media and effects increased with DOMs concentration. Lv *et al*. reported that 5 and 10 mg/L of HA stabilized CeO_2_ NPs in the suspensions by introducing both negative surface charge and steric repulsion and thus enhanced their mobility in the porous media. They also found a similar phenomenon with our results that the mobility of CeO_2_ NPs in the porous media enhanced dramatically with HA concentration increasing^43^. By comparison, influence of HA on NPs transport was more evident than BSA. For experiments with CaCl_2_ as electrolyte, CDC curves were plotted in Fig. 6b,d. Estimated CDC values were 1.0 and 6.3 mM CaCl_2_ for 0.5 and 2.5 mg/L HA, 4.3 and 6.1 mM CaCl_2_ for 5 and 20 mg/L BSA, which were 5.9, 37, 25.3 and 35.9 of that without DOM addition, respectively. CDC values in CaCl_2_ were all significantly smaller than those in NaCl, due to the ability of divalent calcium ions screening negative surface charges was more effective than monovalent sodium, thereby facilitating attachment and deposition. The difference of CDC between Na^+^ and Ca^2+^ at different NOMs were calculated in the range of 8.9–44.8 fold, which were generally lower than the 64 fold difference reported by Grolimund *et al*. for transporting spherical carboxylate latex particle and natural particles through soil^44^. Reference’s results could follow well with the empirical Schulze-Hardy rule, developing from homo-aggregation studies. However, due to presence of DOMs and complexity of surface charge and morphology of NPs and collectors, our results did not follow this rule. investigated the homo-aggregation in presence of DOM in both NaCl solution and synthetic groundwater containing Ca^2+^ and found that DOM dramatically reduced α in both electrolytes^45^. Due to Na^+^, Ca^2+^ and DOMs always coexisted in natural water environment, the results that presence of very low DOMs concentrations caused low NPs α would give valuable suggestions to understand the transport of NPs in natural porous media.

## Methods

### Preparation and characterization of CeO_2_-NPs suspension

Uncoated CeO_2_-NPs were purchased from Sigma-Aldrich. According to the manufacture, it is high purity (99.8%) with primary size of 15–30 nm and specific surface area of 50 m^2^ g^−1^ (BET method). Stock suspensions were prepared using protocol for nanoparticles dispersion provided by CEINT. Briefly, 0.1 g CeO_2_-NPs was weighed into 250 ml beaker containing 200 ml nanopure water (NPW), and then ultrasonicated for 40 min in ice-water mixture, yielding a 500 mg/L stock solution. As shown in Fig. S2, hydrodynamic diameters and zeta potentials of stock solution were roughly 344 nm and 17.5 mV, as measured by dynamic light scattering (DLS)(Zetasizer Nano, Malvern Instruments Ltd). A UV-VIS spectrophotometer (Hitachi U-2810, Japan) was used to characterize the unique surface plasmon absorption band of CeO_2_-NPs. TEM was used to determine its surface morphology and size distribution in solution. All stock suspensions were kept at 4 °C for a maximum of 5 days. Prior to use, these stock suspensions were first well-dispersed by sonicating for 20 min and then diluted to desired concentration.

### Preparation of Silica media and packed columns

Silicate glass beads (potters Industries Inc., Berwyn, PA) with an average diameter of 365 μm were used as model collectors. The beads were successively treated with base (0.1 M NaOH), acid (0.1M HCl, 10% H_2_SO_4_) and DI water before drying (100 °C in air) and storage to remove extraneous materials on surface^46^. Transport experiments were conducted in a vertically oriented borosilicate glass column with an inner diameter of 1.0 cm and length of 10 cm. To ensure uniform packing and excluding air bubbles, glass beads were wet packed into columns using gentle ultrasonic treatment^47^. Beads were kept saturated with NPW before and during packing process. Compacted columns had an effective porosity of approximately 0.41 and total effective volume of 3.22 mL. A nylon filter with pore size of 60 μm was put at column bottom to support the media.

### Column transport experiments

**Figure 7:**
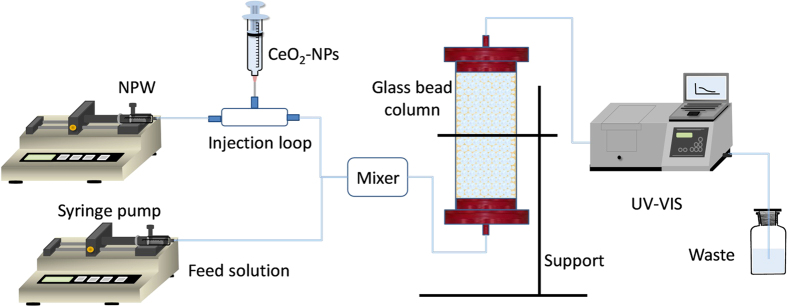
Experimental set-up for column transport experiments.

A dual-pump system was used to inject two feed solutions, including NPW and background electrolyte solution, into column at an upward direction, as shown in Fig. 7. Buffers were not used to eliminate possible impacts on CeO_2_-NPs attachment efficiency. Both solutions were pumped at constant flow rate of 1.0 ml/min by two syringe pumps in most experiments. An injection loop was connected in NPW line for conveniently injecting NPs and eliminating unwanted pressure spikes. Prior to column inlet, these two lines were combined into an in-line mixing chamber (1 mL volume) including a Teflon coated magnetic stir bar (cylinder with length and basal diameter of 12 mm and 3.5 mm) to uniformly mix the solution. To further condition collector surface, at least 10 pore volumes (PVs) of NPW and electrolyte mixture (at desired pH and IS) were pumped through packed column before injecting NPs suspensions. To reduce disturbance of NPs deposition, each packed glass beads was only used for once NPs injection.

Pulse injection mode was used to introduce CeO_2_-NPs into feed solution. After conditioning column, 1.0 mL pulse of 100 mg/L CeO_2_-NPs dispersion was injected into NPW line through the injection loop to minimize aggregation or deposition before entering into column. For most experiments, these solutions were kept at original pH without further adjustment (pH = 5.54), except in pH influencing experiments. By-pass was used in a preliminary experiment for influent CeO_2_-NPs concentration determination, immediately prior to each column run. Real-time influent and effluent concentration of CeO_2_-NPs were monitored online at a wavelength of 319 ± 1 nm by applying a UV-visible spectrophotometer equipped with a temperature controller and a 1 cm flow-through cuvette.

To measure the effect of cationic type and strength on NPs transport, experiments were performed with four chloride salts (KCl, NaCl, CaCl_2_ and MgCl_2_) with cationic concentration range of 0.01–800 mM. To measure pH effect, pH of suspension and feed solution were adjusted to 4.0, 5.54 (original value) and 10.0 by adding appropriate amount of diluted HCl and NaOH. Experiments were performed with NaCl of 0.5–1000 mM. To measure impact of NPs concentration, 15 NPs concentrations in range of 10–200 mg/L without pH adjustment were performed at electrolyte of NaCl (10 and 50 mM) and CaCl_2_ (0.25 and 0.8 mM), respectively. Effect of flow rate was tested by performing experiments at eight different flow rates: 0.5–4.0 mL/min (Darcy velocities of 0.64–5.10 cm/min) with ion strength of 20 mM NaCl and 0.5 mM CaCl_2_, respectively. Two surfactants, SDS and Triton X100, were performed at 0.001–100 mM to see their effects on CeO_2_-NPs transport. Four typical organic matters, including humic acid (HA), tannic acid (TA), bovine serum albumin (BSA) and sodium alginate (SA), were set in range of 0.001–25, 0.001–500, 0.001–2500 and 0.001–2500 mg/L to investigate their impacts with background electrolyte of 50 mM NaCl. Finally, three concentrations of HA (0, 0.5 and 2.5 mg/L) and BSA (0, 5 and 20 mg/L) were performed at different electrolytes of NaCl (0.1–1000 mM) and CaCl_2_ (0.01–100 mM) to investigate existence of organic matters influencing α of NPs.

### Quantitative analysis of transport data

In steady-state flow, transport of CeO_2_-NPs in homogeneous porous media can be described by traditional one-dimensional advection-dispersion equation with a first order kinetic term^44^,


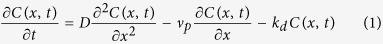


where C(x,t) is aqueous CeO_2_-NPs concentration at position x and time t, ν_p_ is interstitial particle velocity, D is hydrodynamic dispersion coefficient, k_d_ is deposition rate coefficient.

For a pulse-input mode, k_d_ can be calculated based on ratio of CeO_2_-NPs recovered, as follows,


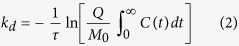


where Q is volumetric flow rate of solution, M_0_ is mass recovery from bypass experiment, τ is average hydraulic retention time (τ = V_pore_/Q).

Particle attachment efficiency (α), defining as ratio of rate particle deposition on a collector to rate of collisions with this collector that has been used, is used to quantitatively interpret transport behavior of nano-particles under different experimental conditions. All α values can be calculated based on colloid filtration theory^48^:


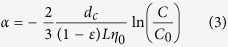


where d_c_ is the mean diameter of spherical collector, ε is porosity of porous medium, L is length of filtering porous media, and C/C_0_ represents the normalized CeO_2_-NPs concentrations in column effluent, which was determined by numerical integration of the area under each measured breakthrough curve of CeO_2_-NPs. η_0_ is the theoretical single collector efficiency of clean-bed, which is composed of three filtration mechanisms, such as particle diffusion, interception and sedimentation. η_0_ values were determined using the empirical equation^49^,


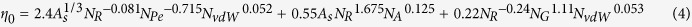


where A_s_ is Happel model parameter, N_R_ is aspect ratio, N_Pe_, N_vdW_, N_A_ and N_G_ are the Peclet number, Van Der Waals number, attraction number and gravity number, respectively, which are calculated from glass bead porosity, particle size, Darcy velocity, particle density, particle hamaker constant, fluid temperature and viscosity. η_0_ was calculated as 0.002122 in this experiment.

Critical deposition concentration (CDC) is commonly defined as electrolyte concentration where α approaches 1.0, and can be obtained from α curve which plots α versus electrolyte concentration on a log-log scale. The approximate CDC can be quantitatively calculated with the following empirical equation:


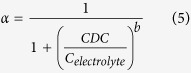


where C_electrolyte_ is background electrolyte concentration (mM), b is slope of log(1/α) as a function of log(C_electrolyte_) in reaction–limited aggregation regime, where C_electrolyte_ ≪ CDC.

## Additional Information

**How to cite this article**: Zhang, Z. *et al*. Transport of cerium oxide nanoparticles in saturated silica media: influences of operational parameters and aqueous chemical conditions. *Sci. Rep.* **6**, 34135; doi: 10.1038/srep34135 (2016).

## Supplementary Material

Supplementary Information
