# Supplementary material for: Transport of cerium oxide nanoparticles in saturated silica media: influences of operational parameters and aqueous chemical conditions
Source: Sci Rep. 2016 Oct 3;6:34135. doi: 10.1038/srep34135 (PMC5046158; doi:10.1038/srep34135)
Supplement: Supplementary Information [file srep34135-s1.pdf]

Supplementary Material

Transport of cerium oxide nanoparticles in saturated silica media: influences of operational parameters and aqueous chemical conditions

Zhaohan Zhang[a,b 1], Peng Gao[a 1], Ye Qiu[a], Guohong Liu[a], Yujie Feng[a*], Mark Wiesner[b]**

a. State Key Laboratory of Urban Water Resource and Environment, Harbin Institute of Technology, No73, Huanghe Road, Nangang District, Harbin 150090, China

b. Center for the Environmental Implications of Nano Technology, Duke University, Durham, North Carolina 27708, United States

* Corresponding authors: yujief@hit.edu.cn (Y.J. Feng); wiesner@duke.edu (Mark Wiesner)

Number of pages: 4

Number of figures: 2

Number of tables: 0

Contents

Fig. S1 Variation of normalized column effluent concentrations with pore volumes number under different concentrations of BSA (a) and HA (b). Results were obtained at 1 mL 100 mg/L CeO2-NPs injection, 50 mM NaCl as background electrolyte, pH 5.54 and flow rate 2 mL/min.

Fig. S2 The UV-Visible spectra (a), size distribution (b), zeta potential (c) and TEM (d) of the CeO2-NPs dispersed suspensions.

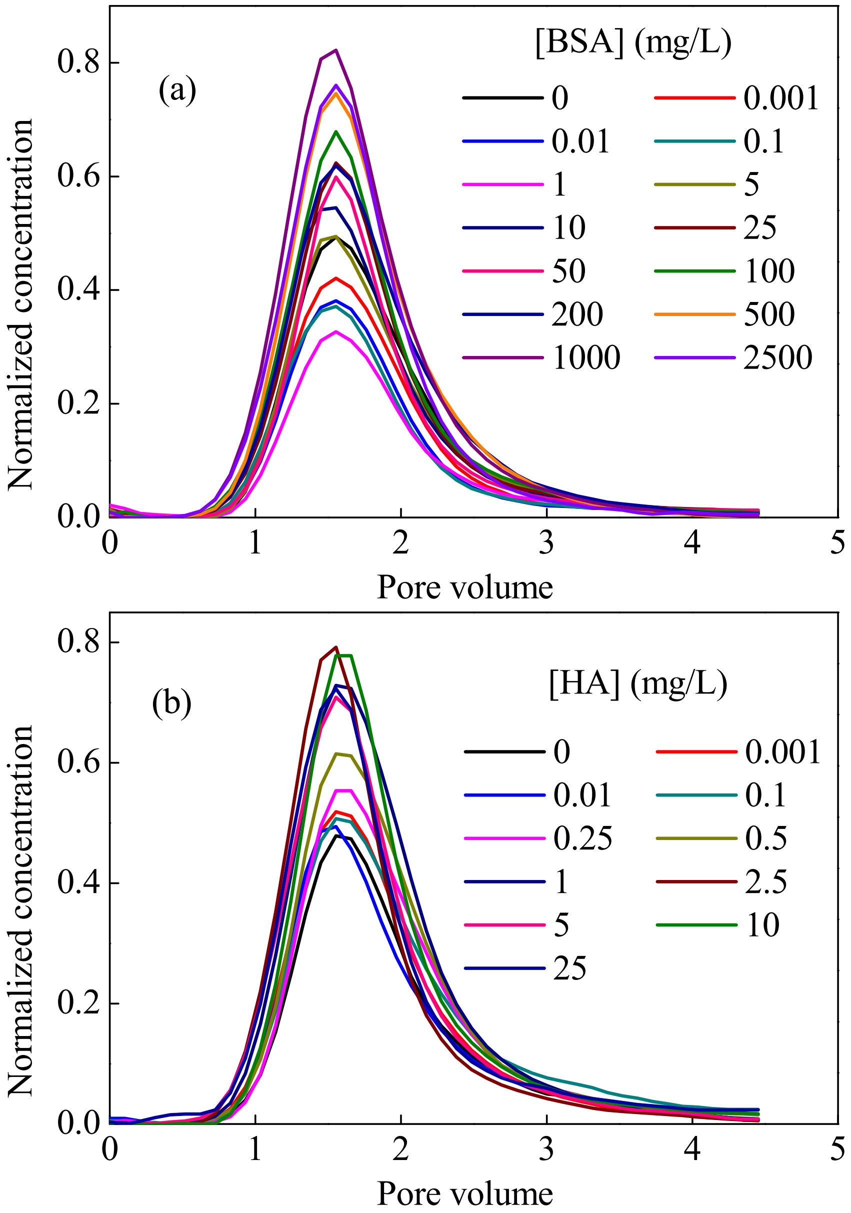

Fig. S1 Variation of normalized column effluent concentrations with pore volumes number under different concentrations of BSA (a) and HA (b). Results were obtained at 1 mL 100 mg/L CeO2-NPs injection, 50 mM NaCl as background electrolyte, pH 5.54 and flow rate 2 mL/min.

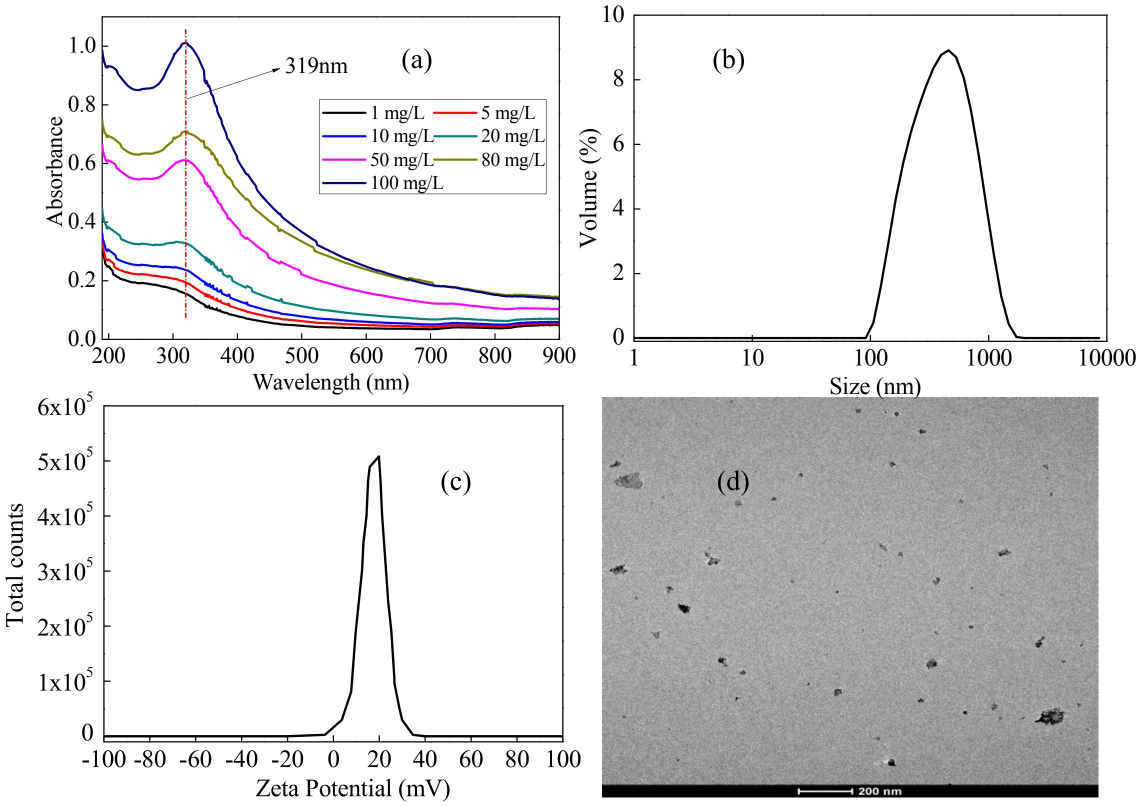

Fig. S2 The UV-Visible spectra (a), size distribution (b), zeta potential (c) and TEM (d) of the CeO_2-NPs dispersed suspensions.
